# Research on the diagnosis model of osteoarthritis based on methylation-related genes using machine learning algorithms

**DOI:** 10.3389/fgene.2025.1595676

**Published:** 2025-08-01

**Authors:** Xu Cui, Houlin Ji, Shengyang Guo, Ju Liu, Linyuan Zhang, Yongwei Jia, Yin Cui, Xiaoxiao Zhou

**Affiliations:** ^1^Department of Orthopedics, Shanghai University of Medicine and Health Sciences Affiliated Zhoupu Hospital, Shanghai, China; ^2^ Jinji Lake Community Health Service Center of Suzhou Industrial Park, Suzhou, China; ^3^Department of Orthopedics, Guangming Traditional Chinese Medicine Hospital, Shanghai, China

**Keywords:** osteoarthritis, methylation, machine learning, diagnostic model, biological functions

## Abstract

**Objective:**

To construct a diagnostic model of osteoarthritis related to methylation genes using machine learning algorithms, and analyze its prognostic value and biological functions.

**Methods:**

The GSE 63695 and GSE162484 datasets including human osteoarthritis (OA) and normal samples were downloaded from the GEO datasets. The microarray chip data of chondrocytes were analyzed using R software to obtain differentially methylated genes. Genes were selected through SVM-RFE analysis and LASSO regression model, and a diagnostic model for OA was established. The performance of the model was assessed by the receiver operating characteristic (ROC) curve. The gene set enrichment analysis of Gene Ontology (GO) and Kyoto Encyclopedia of Genes and Genomes (KEGG) was carried out on the genes incorporated within the model.

**Results:**

An overall 11 DEGs were identified:7 genes were remarkably upregulated and 4 genes were distinctly downregulated. By means of machine learning algorithms, ARHGEF10, ATP11A, NOTCH1, THSD4, NIPA1, SIM2, MAN1C1, ENDOG, CCNC, TAF5, and VPS52 were ultimately incorporated into the model, which could effectively diagnose OA. The area under the curve (AUC) in the datasets GSE 63695 and GSE162484 was 0.96 and 0.93 respectively.

**Conclusion:**

The diagnostic model of methylation-related genes constructed based on machine learning algorithms can effectively identify OA.

## Introduction

Osteoarthritis (OA) is a prevalent degenerative joint disease characterized by cartilage degradation, subchondral bone alteration, and clinical symptoms like pain and mobility limitation. This disease imposes a substantial health burden on individuals, healthcare systems and socioeconomic system (Puig-Junoy and Ruiz Zamora, 2015).Current diagnosis relies heavily on clinical symptoms and imaging, often missing the early disease stage. OA is a progressive and inflammatory disease issue in joint deterioration (Sanchez-Lopez et al., 2022). While traditional biomarkers (e.g., C-reactive protein, procalcitonin, and interleukin-6,etc.) lack specificity, recent studies have explored serum/urine protein markers and radiographic features for early prediction (Collins et al., 2016; Kraus et al., 2018), one study developed a model predicting both pain and radiographic progression using baseline blood and serum markers in OA patients (Kraus et al., 2017), another study performed a multivariable modeling analysis by combining both radiographic and biochemical time-integrated concentration biomarkers that yielded an AUC of 0.712–0.832 to predict future radiographic progression (Hunter et al., 2022). However, these biochemical and radiographic biomarker approaches had their inherent drawback of variability.

To date, genome-wide association studies have identified over 150 genetic risk loci for OA (Boer et al., 2021). Integration of genetic data with molecular profiles of OA-affected tissues accessible at the point of joint replacement surgery can help identify effector genes and their mechanisms of action. It is well-known that the epigenetic modification plays a significant role in directing and maintaining distinctive cellular phenotypes (Mao et al., 2022). Epigenetic patterns are among the first biological changes in disease pathogenesis of OA. Additionally, epigenetic patterns are relatively stable over time (Zhao et al., 2020), suggesting that epigenetic assays may reflect disease-associated changes earlier than traditional protein-based approaches. Studies found that OA holds a distinctive methylation profile (Miranda-Duarte, 2018; Kreitmaier et al., 2024; Hammaker and Firestein, 2018; Ramos and Meulenbelt, 2017). RNA modification serves as important posttranscriptional regulators that participate in the biological processes of eukaryotes and play a pivotal regulatory role in a variety of diseases, such as OA (Jonkhout et al., 2017). One study systematically investigates the crosstalk values for eight-type RNA modifiers in immune landscape during the progression of OA. WDR4 and CFI were distinguished as novel biomarkers and utilized to construct an OA predictive model. Two different RNA modification modes and their connection with immune infiltration were revealed, and a novel scoring system to quantize RNA modification modes in individuals was constructed (Chen et al., 2023).

The study of the methylation state related to OA is conducive to comprehending the progression of the disease and even uncovering its epigenetic markers, thereby facilitating the diagnosis of OA. In this study, we utilized bioinformatics to analyze methylation-related genes of OA patients and construct a diagnostic model, presenting a novel perspective new? for the early identification and treatment of OA.

## Materials and methods

### Source of materials

Using “osteoarthritis.” as the search term, a search and screening were conducted in the Gene Expression Omnibus (GEO) database (https://www.ncbi.nlm.nih.gov/geo/datasets) to obtain the datasets. The high-throughput gene expression dataset GSE63695 was selected and the series of matrix files were downloaded, including healthy hip cartilage from 19 patients with femoral neck fractures, hip cartilage from 16 OA patients, and knee cartilage from 62 OA patients, totaling 97 samples. The samples were divided into a training set of 68 cases and a test set of 29 cases in a 7:3 ratio. Dataset GSE162484 was selected, which contained 10 samples of healthy knee cartilage and OA knee cartilage from 5 cases each, serving as the validation set. The inclusion criteria for the relevant samples are provided in the original dataset [12].

### Data set processing and analysis of differential expression of OA methylated genes

Using the R software (version 4.1.2), the GSE63695 and GSE162484 datasets were preprocessed and normalized through the robust multi-array average algorithm. The “ChAMP” package in the R software was used to conduct differential analysis of methylation sites between OA patients and healthy cartilage in the normalized GSE63695 dataset. The differentially methylated sites that were not annotated to the genome or related to confounding factors were excluded. DMPs (Differentially Methylated Positions, DMPs) within ±2,000 bp of TSS (Transcription Start Site, TSS) were assigned to genes. The screening criteria were adj.P.Val <0.05 & |deltaBeta| ≥ 0.1, where deltaBeta >0.1 represented upregulated methylation sites and <−0.1 represented downregulated methylation sites.

### Machine learning algorithms and feature selection

For the identification of differentially methylated sites, machine learning algorithms were used for feature selection. For the training set, the least absolute shrinkage and selection operator (Lasso) regression method from the R package glmnet (with family set to “binomial”) was employed for data dimensionality reduction and feature selection, thereby obtaining the key methylated site genes related to OA.

### Construction and validation of the diagnostic model

In order to identify the key methylated site genes for OA diagnosis, the genes selected by the previous lasso algorithm were analyzed using the “randomForest” and “SVM-RFE” R implementation algorithms, and ten-fold cross-validation was performed. The λ.1se criterion (the simplest model with the mean error plus one standard deviation) was chosen, and the non-zero coefficient features were retained through iterative optimization using the coordinate descent method. Additionally, to further evaluate the diagnostic performance of the model in other databases, the model was validated on the validation set. The diagnostic performance of the model was evaluated using the ROC curve and AUC.

### Functional enrichment analyses

The relationship of methylation genes included in the prediction model of chondrocytes was analyzed through Cluster Profiler, the relevant methylation sites were screened by constructing a prediction model based on Lasso regression, and gene set enrichment analyses of Gene Ontology (GO) and Kyoto Encyclopedia of Genes and Genomes (KEGG) were performed respectively through the Cluster Profiler package in R package.

### Statistical analysis

All data processing and analysis in this study were completed using R software (version 4.1. 2). The comparison between the two groups of continuous variables was conducted using the Wilcoxon rank sum test, and the statistical significance of normally distributed variables was estimated through the independent t-test. Lasso regression analysis was based on the R package glmnet. A *p* value <0.05 was considered statistically significant.

## Results

### Differential expression analysis of methylation-related genes in OA patients

**FIGURE 1:**
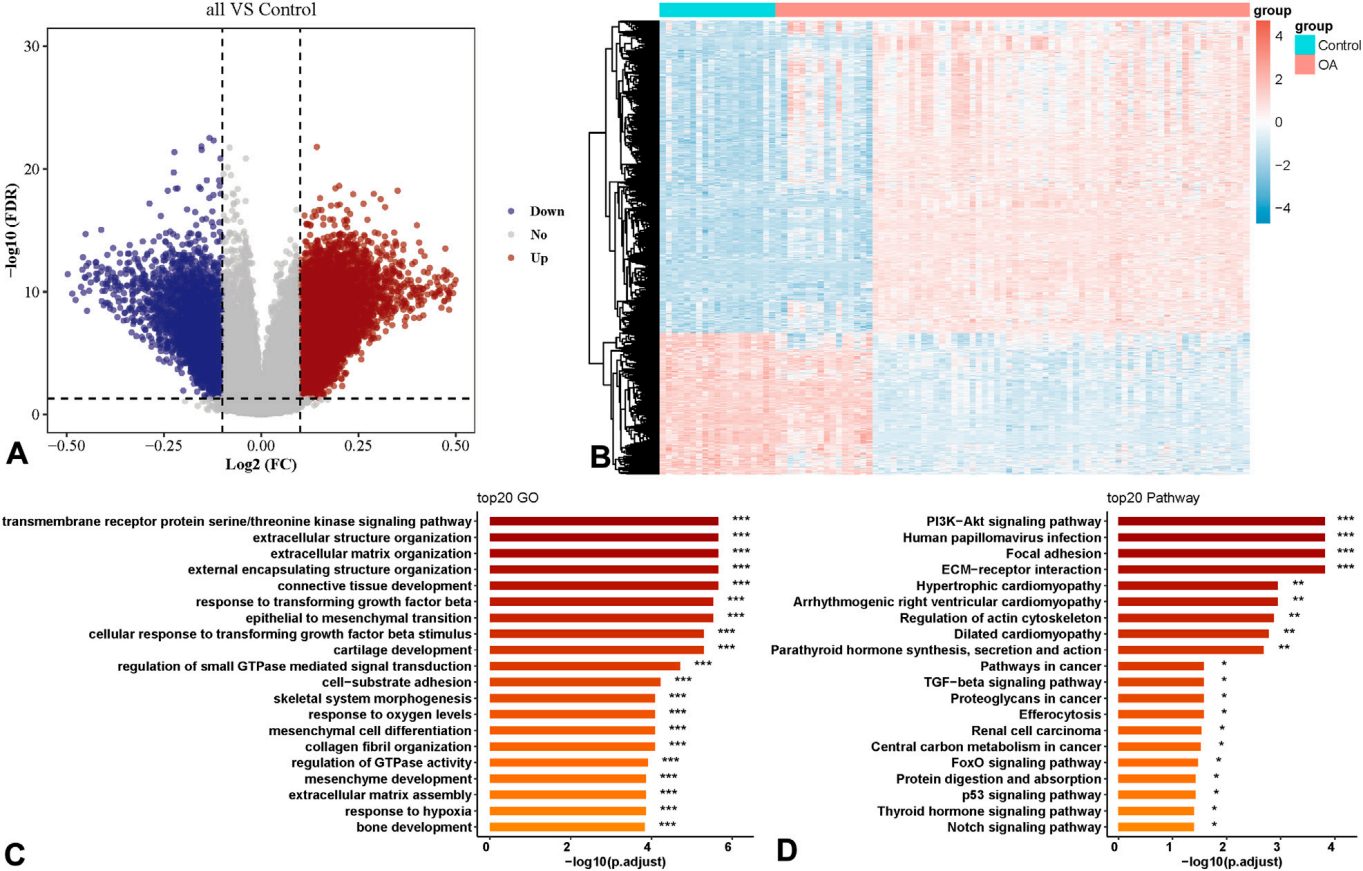
Differential expression analysis of methylation-related genes in Osteoarthritis (OA) patients. **(A)** Volcano plot of differential methylation sites. **(B)** Heatmap of differential methylation sites. **(C)** Functional Gene Ontology (GO) enrichment analysis of differential methylation sites. **(D)** Functional Kyoto Encyclopedia of Genes and Genomes (KEGG) enrichment analysis of differential methylation sites.

By analyzing 113, 659 methylated genes in 21 normal individuals and 96 OA patients in the GSE63695 dataset, 1,574 DEGs were identified, among which 809 were hypermethylated and 765 were hypomethylated (Figures 1A,B). Further analysis of the functional enrichment of DEGs indicated that DEGs were predominantly involved in pathways related to extracellular matrix synthesis, cartilage development, skeletal morphological composition, cytoskeletal regulation, proliferation and apoptosis (Figures 1C,D).

### Constructing an OA diagnosis model based on machine learning

**FIGURE 2:**
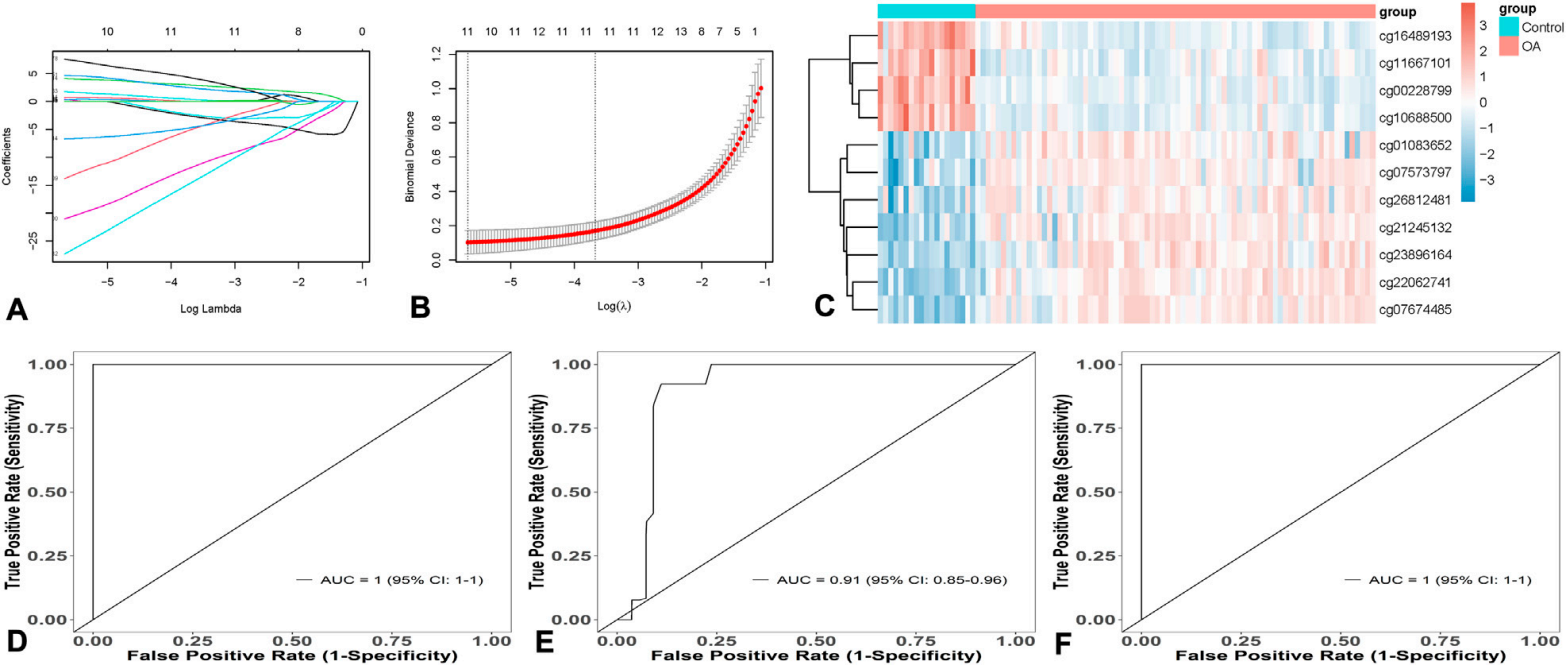
Construction of OA Diagnosis Model Based on Machine Learning. **(A)** and **(B)** Lasso feature selection process diagram. **(C)** Heatmap of individual site grouping. **(D)** Training set. **(E)** 10-fold cross-validation. **(F)** Test Tryout set verification.

In this study, the Lasso algorithm was applied to the OA dataset GSE63695 for data dimension reduction and feature selection (Figures 2A,B). Ultimately, a set of 11 genes with non-zero coefficients was identified: ARHGEF10, ATP11A, NOTCH1, THSD4, NIPA1, SIM2, MAN1C1, ENDOG, CCNC, TAF5, and VPS52. Among them, ARHGEF10, ATP11A, NOTCH1, THSD4, NIPA1, SIM2, and MAN1C1 were highly expressed in OA patients, while ENDOG, CCNC, TAF5, and VPS52 were lowly expressed (Table 1). Heatmaps of individual loci were drawn for the 11 loci obtained from the model training across the entire GSE63695 dataset (Figure 2), showing expression differences in OA. An OA diagnostic model was constructed using the 11 genes with the support vector machine (SVM) algorithm based on the training set, with an AUC of 1.00 (95% CI: 1.00–1.00), a sensitivity of 1.00, a specificity of 1.00, and an accuracy of 1.00 (Figure 2D). The generalization ability of the model was evaluated using a 10-fold cross-validation of the internal validation subset of the original training set, with an AUC of 0.91 (95% CI: 0.85–0.96), a sensitivity of 1.00, a specificity of 0.87, and an accuracy of 0.87 (Figure 2E). The model was also validated using the test set (Figure 2F), with an AUC of 1.00 (95% CI: 1.00–1.00), a sensitivity of 1.00, a specificity of 1.00, and an accuracy of 1.00. The 11 loci demonstrated high diagnostic value.

**TABLE 1:** Eleven methylation site genes were screened based on machine learning algorithms.

	Type	Hip_logfc	Hip_P.Value	Knee_logfc	Knee_P.Value	Gene
cg22062741	Up	0.169294	6.63E-07	0.2537221	1.28E-27	ARHGEF10
cg07674485	Up	0.100332	4.82E-06	0.159867	1.87E-25	ATP11A
cg21245132	Up	0.204269	6.86E-06	0.2522639	8.07E-22	NOTCH1
cg01083652	Up	0.116348	1.63E-05	0.1187086	6.78E-11	THSD4
cg07573797	Up	0.132866	5.12E-05	0.1414844	2.72E-12	NIPA1
cg23896164	Up	0.106955	9.59E-05	0.2190429	6.71E-21	SIM2
cg26812481	Up	0.109517	0.000147749	0.1181323	3.67E-11	MAN1C1
cg00228799	Down	−0.10158	4.12E-10	−0.105762	1.65E-22	ENDOG
cg10688500	Down	−0.10328	6.65E-09	−0.108176	1.01E-21	CCNC
cg11667101	Down	−0.12812	2.64E-08	−0.121101	4.23E-17	TAF5
cg16489193	Down	−0.11016	4.33E-07	−0.14767	2.76E-25	VPS52

### Model verification

**FIGURE 3:**
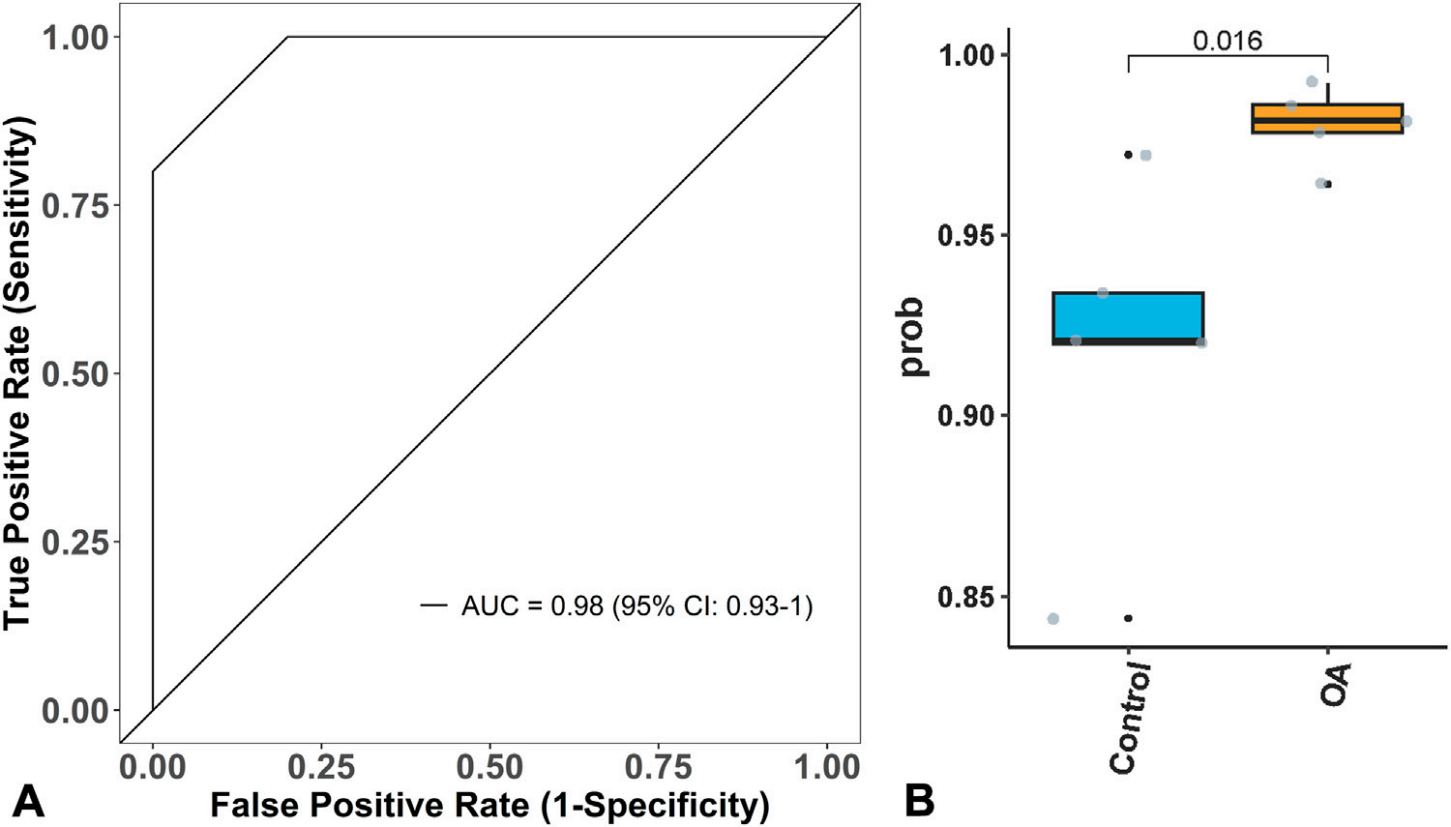
Model verification. **(A)** Model A can effectively verify OA in GSE162484, with an Area under the Curve (AUC) of 0.98. **(B)** the score of Model B for OA is higher than that of normal people.

To further verify the performance of the diagnostic model, it was applied to another independent dataset (GSE162484) from GEO for validation. The ROC curve showed that its AUC was 0.98 (95% CI: 0.93–1.00), with a sensitivity of 1.00, specificity of 0.80, and accuracy of 0.80, as shown in Figure 3A. The model’s predictive group probability score indicated that OA patients had a higher score than healthy cartilage (*p* < 0.05), demonstrating strong discriminatory power of the model, as shown in Figure 3B.

### Biological function prediction

**FIGURE 4:**
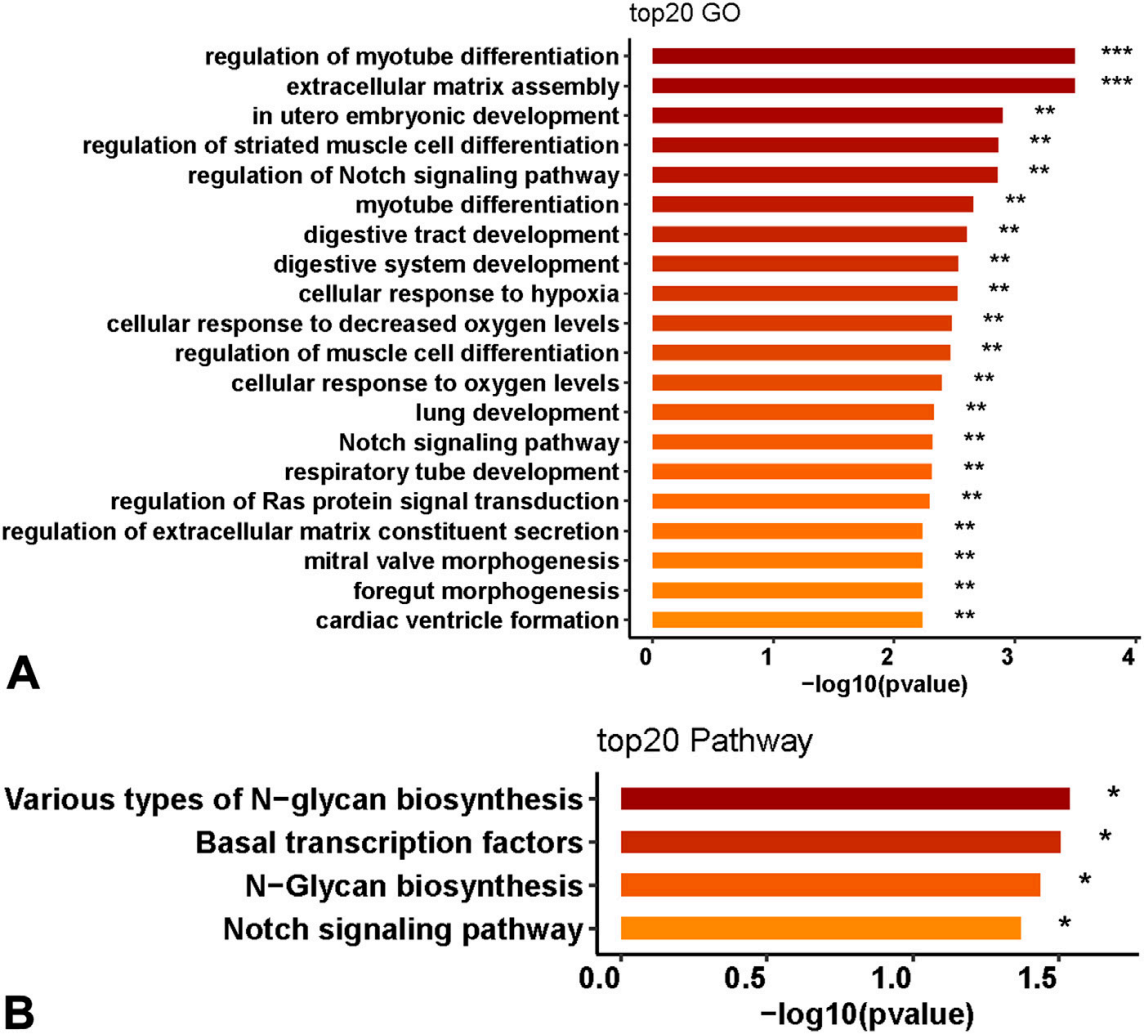
Biological function prediction of 11 genes. **(A)** GO enrichment analysis of the functions of 11 genes. **(B)** KEGG enrichment analysis.

This study further analyzed the functional roles of 11 genes in OA patients and used the R package Cluster Profiler to analyze the functions of GO and KEGG gene enrichment. The results indicated that the 11 genes were mainly involved in the Notch signaling pathway, hypoxia translation regulation of cells, extracellular matrix regulation, and N-glycan synthesis (Figures 4A,B), *p* < 0.05.

## Discussion

Osteoarthritis (OA) is a complex disease that is closely related to genetic and environmental factors. Existing studies have found that epigenetic modification is involved in the pathophysiological process of OA (Valdes and Spector, 2009). Epigenetics describes three main phenomena: 1) Modification of DNA by methylation. 2) Histone side chain modification. 3) Regulation of non-coding RNA (Tammen et al., 2013). They collaborate synergistically to regulate gene transcription in a heritable fashion (Simon and Jeffries, 2017). The classical concept holds that DNA hypermethylation and histone deacetylation are related to gene silencing. DNA hypomethylation and histone acetylation promote gene expression. Existing studies have shown that hypomethylation of the promoter is associated with an increased expression of matrix metalloproteinases, which are involved in the degradation of the cartilage matrix in OA (Miranda-Duarte, 2018). OA possesses a distinctive methylation spectrum. Researching the methylation status related to OA is conducive to understanding the progression of the disease and even discovering its epigenetic markers, thereby aiding in the diagnosis of OA. In this study, bioinformatics analysis was utilized to analyze the differentially methylated genes of OA and normal individuals in the GEO database. Further, machine learning SVM-RFE and Lasso regression algorithms were employed to screen 11 genes to construct a diagnostic model, and the performance of the model was evaluated through the ROC curve. Finally, the biological functions of the methylated genes in the model and their diagnostic value for OA were analyzed.

The pathogenesis of OA is still unclear. Its main pathological characteristics include the degradation of extracellular matrix of chondrocytes and apoptosis of chondrocytes. OA is usually assumed to be caused by non-inflammatory factors; that is, a series of mechanical stresses that destroy the cartilage. Existing studies shown some associated inflammatory factors have also been shown to contribute to OA development, allowing inflammatory cells to infiltrate the synovium (Nedunchezhiyan et al., 2022). Moreover, inflammatory cytokines play an essential role in the progression of OA by stimulating the production of matrix metalloproteinases and thus increasing matrix degradation (Woodell-May and Sommerfeld, 2020). However, recently, studies have shown that DNA methylation is involved in the pathological mechanism of OA (Rushton et al., 2014). An increasing number of studies have focused on the effects of nucleic acid site changes on the cell function and even body activities, wherein RNA modification plays a critical role. One study highlights widespread epigenetic markers for cartilage degeneration linked to a large spectrum of biological pathways, including apoptosis and neuronal development, which reveal large similarities in the epigenetic signature of OA across sexes, but also finds a number of sex-specific markers, thus providing enhanced insights into the OA related epigenetic signature in cartilage (Kreitmaier et al., 2024). In cartilage, epigenome-wide association studies (EWAS) have been conducted to compare macroscopically intact (low-grade) and degraded (high-grade) OA cartilage samples to study epigenetic markers of cartilage degeneration (den Hollander et al., 2015). DNA methylation studies have generated valuable profiles of OA tissues (Katsoula et al., 2022), such as cartilage, synovium (Kreitmaier et al., 2022) and subchondral bone (Zhang et al., 2016). In this study, the expression differences of methylated genes between OA patients and normal individuals were analyzed. The results indicated that 7 genes were hypermethylated and 4 genes were hypomethylated in OA patients, indicating that methylated genes play a significant role in the disease process of OA.

Currently, the diagnosis of OA in clinical settings rely on the clinical symptoms and imaging manifestations of patients, which has the drawbacks of subjectivity and inability to achieve early diagnosis. Machine learning currently forms the commonly used novel statistical method for analyzing large-scale data in the medical field, including decision trees, SVM-RFE, deep learning, and others. It can construct predictive models by screening clinical characteristics and sequencing data (Bagante et al., 2019). The study by Liang and his colleagues constructed an m7G-related scoring model, used two different machine learning methods to avoid the one-sidedness of screening methods, which can significantly differentiate patients with OA, and correlated it with different statuses of the immune microenvironment, based on which a diagnostic model to diagnose patients with OA was constructed (Hao et al., 2023). By developed peripheral blood DNA methylation-based machine learning models for prediction of knee OA progression in the OA Biomarkers Consortium cohort and validated the findings in two independent cohort, which suggest that pain and structural progression share similar early systemic immune epigenotypes (Dunn et al., 2023). In our study, the methylation sequencing data of OA and normal individuals from the GEO database were used. By integrating the machine learning algorithms SVM-RFE and Lasso regression, 11 OA-related methylation genes were eventually screened out to build a model. The AUC of the ROC in the dataset GSE162484 was 0.98, suggesting that this model can effectively diagnose OA and provides new insights for the early diagnosis of OA.

GO and KEGG functional enrichment analyses of the genes in the model revealed that 11 genes were mainly involved in the Notch signaling pathway, cellular regulation of hypoxia translation, extracellular matrix regulation, and N-glycan synthesis. The Notch signaling pathway is closely related to the proliferation, differentiation, and apoptosis of chondrocytes and may mediate the occurrence and development of osteoarthritis through immune inflammation. The Notch signaling pathway is also involved in the synthesis and decomposition of the cartilage matrix (Wang et al., 2023). The translation regulation of cells under hypoxia, especially hypoxia-inducible factors, is a key regulatory gene for maintaining cartilage homeostasis. It takes part in cartilage chondrogenesis metabolism and autophagy apoptosis, thus playing a significant role in the pathological development of OA (Guo et al., 2022).

The regulation of the extracellular matrix is a key factor in the occurrence and development of OA. With the increase of age, the loss of regulation of the molecular pathway affects the dynamic balance of the extracellular matrix in cartilage, leading to the destruction of the cartilage structure (Lotz and Loeser, 2012). Proteoglycan is an important part of the extracellular matrix and plays an important role in the pathological process of OA (Coryell et al., 2021). Consequently, these functions exert a crucial role in the OA pathological process, further suggesting that the relevant methylation genes are implicated in the pathological development of OA. Hence, this model possesses favorable diagnostic value and significance.

Nevertheless, this study presents certain limitations. Firstly, as the data source was a public database, the sample number of control group and OA group didn’t equivalent, and input mistakes could not be determined. Secondly, this study merely analyzes the data within the GEO database and lacks clinical validation. The study uses only two GEO datasets with relatively small sample sizes, particularly in the validation set. Thirdly, the data in the GEO database lack clinical grading data, thereby making it impossible to predict the severity of OA at different levels. Therefore, a multi-center large-sample dataset is required to further enhance the prediction model. A generalizability to broader OA populations and the need for further validation in larger, prospective cohorts. Furthermore, additional experiments like flow cytometry and single-cell sequencing are still required to elucidate the mechanism in detail. However, to reveal causal links between OA and cartilage methylation, the results of our study need to be further verified in cell or animal/human experiments, which will be critical direction for future research and translate into clinical implementation.

## Conclusion

In this study, the OA diagnostic model was constructed based on the machine learning SVM-RFE and Lasso regression algorithms with the GEO database, which can effectively identify OA patients. Furthermore, the genes in the model have guiding significance for the prognosis of OA patients. Our study highlights epigenetic markers for cartilage degeneration linked to a large spectrum of biological pathways, including the Notch signaling pathway, and cellular regulation of hypoxia translation, etc., providing enhanced insights into the OA related epigenetic signature in cartilage and a theoretical basis for the early identification and timely treatment of OA in clinical practice, thereby improving the care of adults with OA.

## Data Availability

The datasets presented in this study can be found in online repositories. The names of the repository/repositories and accession number(s) can be found in the article/supplementary material.

## Abbreviations

OA, Osteoarthritis; ROC, Receiver Operating Characteristic; GO, Gene Ontology; KEGG, Kyoto Encyclopedia of Genes and Genomes; AUC, Area under the Curve; GEO, Gene Expression Omnibus; SVM-RFE:Support Vector Machine Recursive Feature Elimination.
